# Genotype-by-environment interactions and local adaptation shape selection in the US National Chip Processing Trial

**DOI:** 10.1007/s00122-024-04610-3

**Published:** 2024-04-10

**Authors:** Husain I. Agha, Jeffrey B. Endelman, Jessica Chitwood-Brown, Mark Clough, Joseph Coombs, Walter S. De Jong, David S. Douches, Charles R. Higgins, David G. Holm, Richard Novy, Marcio F. R. Resende, Vidyasagar Sathuvalli, Asunta L. Thompson, G. Craig Yencho, Lincoln Zotarelli, Laura M. Shannon

**Affiliations:** 1 Department of Horticultural Science, University of Minnesota, Saint Paul, MN USA; 2 Department of Plant & Agroecosystem Sciences, University of Wisconsin-Madison, Madison, WI USA; 3 Department of Horticulture and Landscape Architecture, Colorado State University, Fort Collins, CO USA; 4 Department of Horticultural Science, North Carolina State University, Raleigh, NC USA; 5 Department of Plant Soil and Microbial Sciences, Michigan State University, East Lansing, MI USA; 6 School of Integrative Plant Science, Cornell University, Ithaca, NY USA; 7 Potatoes USA, Denver, CO USA; 8 Small Grains and Potato Germplasm Research, USDA-ARS, Aberdeen, ID USA; 9 Horticultural Sciences Department, University of Florida, Gainesville, FL USA; 10 Hermiston Agricultural Research and Extension Center, Oregon State University, Hermiston, OR USA; 11 Department of Plant Sciences, North Dakota State University, Fargo, ND USA

## Abstract

**Key message:**

We find evidence of selection for local adaptation and extensive genotype-by-environment interaction in the potato National Chip Processing Trial (NCPT).

**Abstract:**

We present a novel method for dissecting the interplay between selection, local adaptation and environmental response in plant breeding schemes. Balancing local adaptation and the desire for widely adapted cultivars is challenging for plant breeders and makes genotype-by-environment interactions (GxE) an important target of selection. Selecting for GxE requires plant breeders to evaluate plants across multiple environments. One way breeders have accomplished this is to test advanced materials across many locations. Public potato breeders test advanced breeding material in the National Chip Processing Trial (NCPT), a public–private partnership where breeders from ten institutions submit advanced chip lines to be evaluated in up to ten locations across the country. These clones are genotyped and phenotyped for important agronomic traits. We used these data to interrogate the NCPT for GxE. Further, because breeders submitting clones to the NCPT select in a relatively small geographic range for the first 3 years of selection, we examined these data for evidence of incidental selection for local adaptation, and the alleles underlying it, using an environmental genome-wide association study (envGWAS). We found genomic regions associated with continuous environmental variables and discrete breeding programs, as well as regions of the genome potentially underlying GxE for yield.

**Supplementary Information:**

The online version contains supplementary material available at 10.1007/s00122-024-04610-3.

## Introduction

Variation in environment across a species’ geographic range can result in selection for local adaptation, leading to foreign populations having lower fitness than populations in their home environment. There has been extensive research both into patterns of local adaptation in natural populations (Ågren and Schemske 2012; Alberto et al. 2013; reviewed in Shaw and Etterson 2012; Sork 2018), and modern crop species as it relates to efforts to address climate change (Howden et al. 2007; Takeda and Matsuoka 2008). However, only large-effect genes and alleles that underlie environmental adaptation on a broad scale have been mapped, such as *StCDF1* and *ZmCCT*, major genes contributing to long-day adaptation in potato and maize, respectively (Kloosterman et al. 2013; Hung et al. 2012). Many commonly used selection mapping tools depend upon large differences in allele frequencies between populations to find statistical evidence for regions under selection (e.g.,Beaumont and Nichols 1996; Beaumont and Balding 2004; Foll and Gaggiotti 2008), generally relying on F_ST_ (Wright 1949) or similar statistics. These tools are poorly suited to study polygenic adaptation because such adaptation is usually a result of many, small allele frequency changes dispersed across the genome (Berg and Coop 2014). Local adaptation is a complex trait, and selection mapping is unlikely to differentiate genomic signals of local adaptation from drift (Hancock et al. 2010; Uricchio et al. 2019). Identifying new tools and models to understand the relationship between artificial selection and response to environment will aid breeders in establishing lines adapted to certain environments.

Potato (*Solanum tuberosum* L.) is a useful model for studying the interaction between environmental adaptation and artificial selection. Firstly, public potato breeders incorporate untested germplasm from different programs to make up much of their field in the 1st year of selection, which greatly reduces drift between populations and contributes to the limited hierarchical population structure in the US commercial potato (Bali et al. 2018; Hirsch et al. 2013; Pandey et al. 2021). Secondly, while major targets of selection for commercial application are generally the same among programs, environments between programs differ greatly. Lastly, potatoes are clonally propagated, which allows multi-environmental and multi-year observations of identical genotypes without inbreeding.

Potato breeding generally involves growing clonally propagated, F1 individuals at a single location for 2 years and a second, geographically proximal location in the 3rd year before clones are entered in multi-location trials. Typically, only ~ 0.1% of the individuals tested in the first field year are selected to continue beyond the third field year, based almost entirely on recurrent phenotypic selection with limited replication; in most programs, only a single individual per clone is used in the first field year. There is extensive GxE for both yield and quality traits in potato (Affleck et al. 2008; Yildirim and ÇaliŞkan 1985). The intense selective pressure in a relatively small geographic range combined with the strong effect of GxE on important traits may lead to unintentionally selecting genotypes that are locally adapted. However, because programs often exchange material before the first field year, the underlying genetic variants giving rise to local adaptation are likely to be transient, i.e., no one variant is likely to be repeatedly selected across generations (Yeaman 2015). This might especially be the case as clones that perform well across multi-environment trials tend to be overrepresented as parents in subsequent years, leading to artificially inflated gene swamping, a phenomenon where gene flow reduces the frequency of locally adapted alleles (García-Ramos and Kirkpatrick 1997; Haldane 1956; Kirkpatrick and Barton 1997; Polechová 2018; Polechová and Barton 2015). Consequently, we expect only locally adapted alleles that are conditionally neutral, showing no negative effect outside of the home environment, and that are present in the most successful lines to persist year to year (Anderson et al. 2013). We further expect variants that meet these criteria to be exceedingly rare. Understanding the dynamics among these antagonistic processes, selection for locally adapted alleles and gene swamping from dissimilar environments, in potato will give us insight into the relative contribution of local adaptation to early variety development in potato breeding programs, which may influence selection strategies to maximize genetic gain within and between environments.

To identify loci underlying local adaptation in potato, we interrogated data from the US National Chip Processing Trial (NCPT), where advanced chipping clones from public breeding programs in the US are tested across diverse environments. Clones are initially submitted to the NCPT in their fourth field year. Most clones are only included in the NCPT once, though promising lines may be tested in subsequent years. Under this selection scheme, we would expect clones with an advantage in their home environment to potentially outperform more generalist clones before entering the NCPT. This may lead to a relatively high frequency of the genetic variants underlying local adaptation in the genotypes being submitted to the NCPT, even if those variants are maladapted in many target environments.

We scanned the genome for associations between allele frequency and different environmental measurements. We used continuous environmental measurements during selection (i.e., the 3 years prior to a clone’s entry into the NCPT in its home site) as quantitative response variables and discrete programs as case-control response variables in what is called ‘environmental genome-wide association studies’ or envGWAS (Lasky et al. 2023; Li et al. 2019; Rowan et al. 2021; see also Lasky et al. 2015; Turner et al. 2010). For these analyses, we assumed that a clone’s presence in the NCPT is evidence of strong relative performance in its home environment, as only strongly performing clones are submitted to the NCPT. It is important to note that we do not expect the genetic markers used in our GWAS models to affect the environment, i.e., the environment is independent of allele frequency. envGWAS utilizes a ‘reverse regression’ technique, where the independent variable is used as the response variable and the dependent variable is used as the regressor. Reverse regression generally violates a key assumption of linear regression, that the independent variables are measured without error while the dependent variable is measured with error, but this assumption is not likely to be violated in envGWAS when using high-quality genetic markers. While this method can lend insight into what genomic regions may be underlying local adaptation, generally, envGWAS as described above does not take into account any phenotypic information. envGWAS, therefore, cannot lend insight into *which* traits may be responding to selection between environments, only *whether* selection for environmental response occurred.

Another key assumption of envGWAS is that it leverages hundreds (or thousands) of generations of selection in order to find evidence of local adaptation. For many modern crop breeding pipelines, this would make envGWAS inappropriate, as there are too few generations to establish a strong enough signal of selection considering the polygenic nature of local adaptation. However, because potato breeding employs such intense selection in a single environment coupled with the use of clones across years, it presents a unique opportunity to identify alleles interacting with the environment. Unlike in natural populations, where ordinarily selection coefficients are relatively weak, in the potato breeding pipeline presented, only ~ 1 in 1000 genotypes tested in a small geographic region will be selected to move forward to the NCPT. Additionally, because potato is clonally propagated during this selection, both the additive and non-additive genetic effects underlying local adaptation will be preserved (i.e., the genetic gain for local adaptation is governed by the broad-sense heritability rather than the narrow-sense). Together, this intense selection in a small geographic region combined with a clonal selection scheme should result in a similar response to selection for local adaptation compared to much longer timescales in wild or landrace populations.

Aside from exploring these data for associations between different aspects of the selection environment and allele frequencies, we looked for loci associated with genotype-by-environment interactions (GxE) for yield in the trial environments. To do so, we calculated reaction norms by regressing yield onto environmental measurements. Differences in the slope of the reaction norms between genotypes indicate GxE, and we map regions of the genome underlying GxE across specific environmental gradients by building GWAS models for the slope of these reaction norms (Tétard-Jones et al. 2011). Taken together, these analyses can lend insight into the unintended effect of selection on local adaptation and GxE in the NCPT, and can be used to intentionally breed ecotypes for specific environments.

Finally, we looked for associations between allele frequency and submission year to understand how allele frequencies may be changing over time using Generation Proxy Selection Mapping (GPSM; Decker et al. 2012; Rowan et al. 2021; Walsh and Lynch 2018). GPSM identifies regions of the genome under directional selection by associating allele frequency with an individual's generation (or a proxy, thereof). Here, we use the year a clone was initially submitted to the NCPT as its generation. We use GPSM to look for regions of the genome that is both under directional selection and associated with aspects of the environment identified by envGWAS. Combining these results allows us to understand if variants underlying local adaptation are increasing in frequency over the course of the trial period tested. One important caveat for this analysis is that the parents of a clone submitted in any given year are generally not from the immediately preceding generation and, in fact, may not even be from the same generation. This type of crossing scheme complicates the interpretation of ‘generation’ in potato and may limit the power of GPSM to detect signals of directional selection.

Though climate change increases temperature generally, it also makes differences between environments more pronounced, e.g., prolonged drought in one region with simultaneous flooding in another (Trenberth 2005). As our growing environments become more disparate, there may need to be an increased focus on locally adapted cultivars. This is especially true in the global south, where climate change will be felt most strongly (Mendelsohn et al. 2006). Even ignoring predicted changes to the climate, breeders may be leaving potential genetic gains on the table by focusing on broadly performing lines (Ewing et al. 2019). While this method might be justified for other crops, where the cost of regional breeding programs exceeds the benefit, potato breeding in the US is almost entirely carried out by regional, public breeders. Finding genomic regions that underlie local adaptation will help these breeders make progress in selecting for environment-specific germplasm under either scenario. Here, we first show that envGWAS can be used to find genomic variants that are potentially locally adapted, both along measurable environmental gradients and to specific geographic ranges. We then show that similar methods can be used to look for regions of the genome that may be responsible for GxE to specific environmental variables. Finally, we scanned the genome for regions that are under directional selection to see if these overlap with those identified in our previous analyses. These results present a starting point for generating hypotheses about the molecular basis of local adaptation and its influence on early generation variety development in potato and beyond.

## Materials and methods

### National trial data

**Fig. 1:**
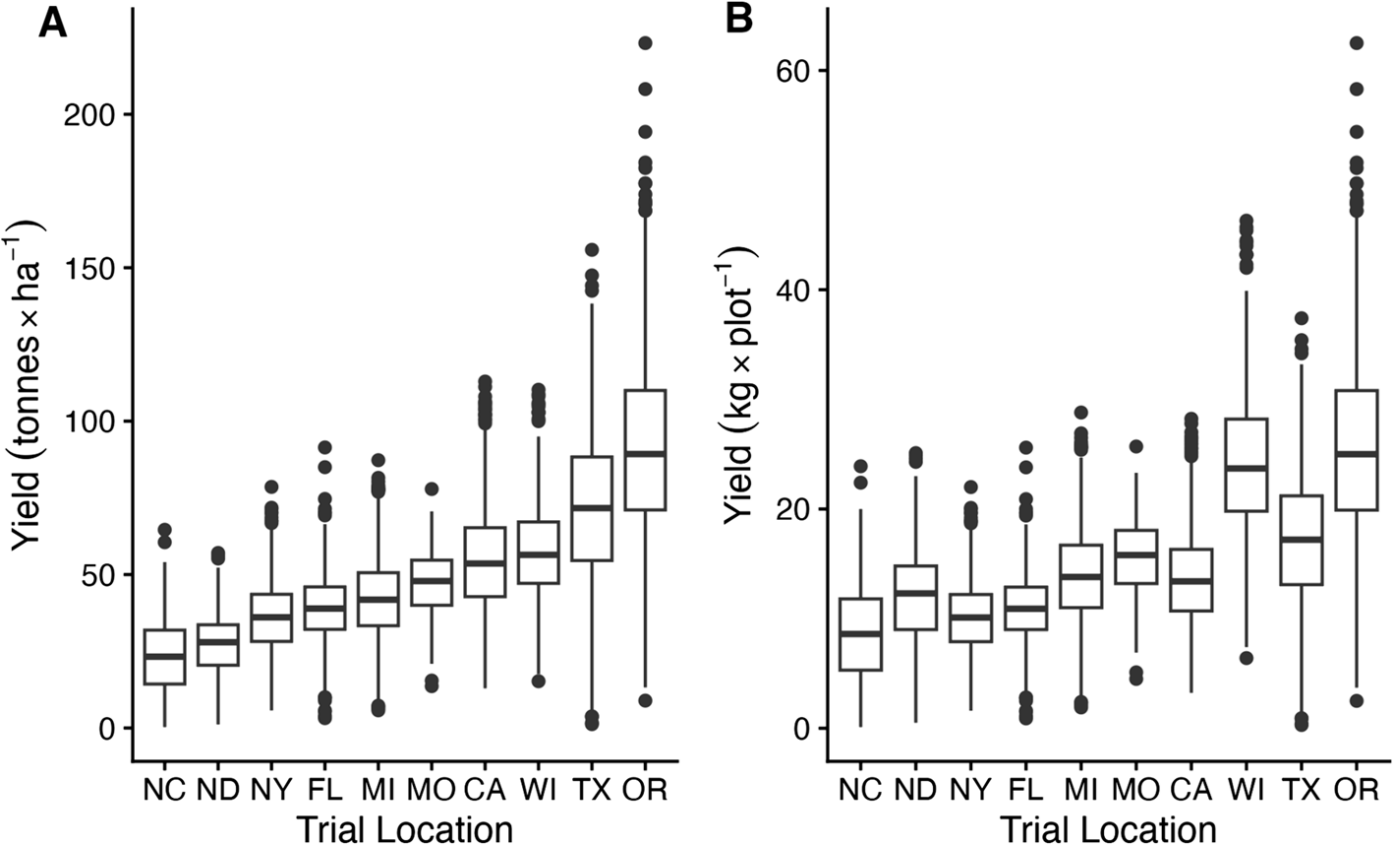
Box and whisker plot of yield (**A**: ton × hectare^−1^; **B**: kg × plot^−1^) for each trial location from 2014 to 2020. Black lines show the median yield in each trial. Boxes show the 1st and 3rd quartiles. Whiskers extend up to 1.5*interquartile range or to the range of the data. Points show outlier observations outside whisker borders. Note that neither plot size nor number of plants are standard across trial locations

Yield data from 2010 to 2022 for up to ten trial locations per year (California, Florida, Michigan, Missouri, New York, North Carolina, North Dakota, Oregon, Texas, and Wisconsin; Table 1) were downloaded from the NCPT database (https://potatoesusa.medius.re). In California, 18 seed pieces were planted in two-row plots with in-row spacing of 0.17 m and 0.8 m between rows, while all other locations used single-row plots of 15 seed pieces, with in-row spacing of 0.2 m–0.3 m and between-row spacing of 0.8 m–1.4 m. Trial management varied across locations, with the intent of mimicking local commercial growing conditions. Total yield was calculated from the weight of all tubers in each plot (kg) and varied widely between trial sites (Fig. 1A). Yield per area was calculated as the total yield (kilograms) divided by the plot size (hectares; Fig 1B). We partitioned the genotypic variance for yield by modeling genotype (g), genotype-by-year (gY), genotype-by-location (gL) and genotype-by-year-by-location (gYL) as random effects and year (Y), location (L) and year-by-location (YL) as fixed effects:

$$\gamma = \mu + {\text{Y}} + {\text{L}} + {\text{YL}} + {\text{ g}} + {\text{gY}} + {\text{gL}} + {\text{gYL}} + \varepsilon ,$$

**Table 1:** Coordinates and nearest cities for trial locations in the National Chip Processing Trial as well as coordinates for nearest weather station reported by the National Weather Service. Fields were rotated annually, so coordinates for trial sites are approximate

State	Nearest city	Trial site coordinates		Weather station coordinates	
		Latitude	Longitude	Latitude	Longitude
CA	Bakersfield	35.26	− 118.88	35.43	− 119.06
FL	Hastings	29.68	− 81.43	29.77	− 81.47
MI	Lakeview	43.35	− 85.17	42.88	− 85.52
MO	Charleston	36.93	− 89.38	37.23	− 89.58
NC	Plymouth	35.87	− 76.65	35.85	− 77.03
ND	Hoople	48.53	− 97.62	47.95	− 97.18
NY	Ithaca	42.43	− 76.39	42.49	− 76.46
OR	Hermiston	45.81	− 119.28	45.83	− 119.26
TX	Dalhart	35.97	− 102.73	36.02	− 102.55
WI	Hancock	44.12	− 89.54	44.12	− 89.54

With mean μ and error ϵ using R/lme4 (Bates et al. 2015) in R (v4.1.0; R Core Team 2021).

These analyses used three, overlapping datasets. For the genetic variance decomposition, we used all recorded yield records from 2010 to 2022, regardless of whether there was an associated genotype. This resulted in 22,592 non zero yield observations for 1479 unique clones. For the envGWAS, we used all clones with genotype data, excluding checks and named varieties, resulting in 840 unique genotypes. For the GWAS on the regression lines of yield onto environmental measurements, referred to as ‘regression GWAS’ for simplicity, we included checks and named varieties and had phenotype and genotype data for 870 unique genotypes and 16,881 non zero yield records. To account for differences in plot spacing and number of individuals per plot in the regression GWAS, we normalized phenotypic data by dividing each plot yield by the mean yield within each location-year pair (hereafter referred to simply as ‘trial’). Normalized values were then log-transformed to give the relative performance of each clone within each trial centered on zero.

All clones included in the analyses had previously been genotyped using the potato Infinium SNP array, which has evolved through four versions (Felcher et al. 2012; Vos et al. 2015). Tetraploid genotype calls (coded 0–4) were made using a normal mixture model with R/fitPoly (Zych et al. 2019), and data from earlier versions of the array were imputed up to the current version (V4), for a total of 15,133 markers. Imputation was done with R/randomForest (Breiman 2001; Liaw and Wiener 2002) and 100 classification trees, using default parameters. For each imputed marker, the 100 closest markers based on the DMv6.1 reference genome (Pham et al. 2020) were used as predictor variables. We filtered out SNPs with minor allele frequencies less than 0.01, resulting in 14,838 polymorphic SNPs.

We used principal component analysis (PCA) to look for evidence of population structure. Principal components (PCs) were calculated using the *prcomp* function in R. The first two PCs were plotted using the R/ggbiplot (Figure S1; Vu 2011).

### Environmental variables

We collected precipitation and temperature data from the National Weather Service (weather.gov), using the weather station nearest to the trial (Table 1) and selection (Table 2) sites. Missing data was imputed using information from the next proximal station. Environmental variables were collected over the growing season, which varied by program (Table 3). Maximum daily temperature (maxTemp) and minimum daily temperature (minTemp) were collected as averages over the growing season. Precipitation was collected as the sum over the growing season. These variables were then used in two ways: We averaged the environmental variables over the 3 years prior to their entry into the NCPT at the *selection site*, representing the environment during early selection, and we averaged the variables over the growing season *within each trial*. We chose the 3 years prior to a genotype’s entry into the NCPT as the selection environment as breeders generally first submit their most promising material to the NCPT in the fourth field year (e.g. after 3 years of selection have occurred in the selection site).

**Table 2:** Coordinates and nearest cities for selection locations of material submitted to the National Chip Processing Trial as well as coordinates for nearest weather station reported by the National Weather Service. Fields were rotated annually, so coordinates for selection sites are approximate

State	Nearest city	Selection site coordinates		Weather station coordinates	
		Latitude	Longitude	Latitude	Longitude
CO	San Luis Valley	37.71	− 106.14	37.69	− 106.31
ID	Aberdeen	42.95	− 112.83	43.86	− 111.28
ME	Presque Isle	46.65	− 68.01	46.68	− 68.05
MI	Douglass Township	43.35	− 85.18	42.88	− 85.52
NC	Plymouth	35.87	− 76.65	35.85	− 77.03
ND	Hoople	48.53	− 97.62	47.95	− 97.18
NY	Ithaca	42.43	− 76.39	42.49	− 76.46
OR	Hermiston	45.81	− 119.28	45.83	− 119.26
TX	Dalhart	36.08	− 102.60	36.02	− 102.55
WI	Hancock	44.12	− 89.54	44.12	− 89.54

**Table 3:** GWAS results from case–control phenotypes for selection sites. Chr: chromosome. PVE (%): percent variance explained. Effect: effect size estimate. Effect size refers to the change in the likelihood a variety came from the associated program relative to all other programs

Trait	Marker	Chr	Position	LOD Score	PVE
ME	PotVar0121031	4	13,501,726	6.56	3.12
MI	PotVar0099575	1	87,260,565	5.20	3.01
MI	solcap_snp_c1_14388	9	666,830	6.17	2.90
MI	solcap_snp_c1_2187	11	3,304,705	6.77	3.68
NY	ST4.03ch01_73188644	1	74,255,819	6.69	2.36
NY	ST4.03ch04_25067831	4	13,780,821	6.99	0.19
NY	PotVar0082693	4	20,100,066	9.32	1.96
NY	PotVar0119445	6	43,717,362	8.74	1.07
NY	ST4.03ch06_52872814	6	52,955,662	10.13	1.68
NY	solcap_snp_c2_4393	9	13,198,011	5.11	1.62
NY	ST4.03ch10_4547390	10	4,204,396	5.79	1.14
WI	ST4.03ch07_42076084	7	43,790,278	5.44	2.78

### GWA analyses

We conducted environmental genome-wide association studies (envGWAS) using R/GWASpoly package (Rosyara et al. 2016). We tested for SNPs associated with local adaptation to a general environment in two ways. First, we built an envGWAS model with each selection site as a case–control response variable, which we called the discrete program test. To reduce the effect of sampling error, we only tested for associations with discrete programs if a program submitted at least 84 genotypes to the trial during this period (10% of all genotypes tested), leaving Maine (158 genotypes), Michigan (250 genotypes), New York (86 genotypes), and Wisconsin (141 genotypes). We justify using binary traits in our association analyses by recognizing the linear model as a first-order Taylor approximation to the generalized linear model and the robustness of linear models to misspecification (Zhou et al. 2013). Secondly, we build models using latitude and longitude of the selected site as a quantitative response variable. We used latitude and longitude as responses in the model as they are strongly correlated with other environmental variables (e.g. latitude: daylength and temperature, longitude: precipitation, elevation, and soil pH). Latitude and longitude represent “general” environments which allow us to identify potential SNPs correlated with one more of these environmental variables, as well as their interactions.

We tested for SNPs associated with adaptation to specific environmental variables by using the environment during early selection as the response variable in the GWAS models. The 3-year average of minTemp, maxTemp, and precipitation at the selection sites were used as continuous response variables. These serve as the environment during early selection. We then built linear models in the *lme4* package and calculated the simple slope for each genotype, using yield as the response variable and the environmental variables (minTemp, maxTemp and precipitation) during the trial as the explanatory variable,

$$\gamma = \mu + g + E_{i} ,$$

where *E*_*i*_ is the measurement of the environmental variable *i*. The simple slope was estimated for each genotype in all three environmental variables separately and used as the response variable in GWAS. Finally, we used the 1st year a clone was submitted to the NCPT as a proxy for its generation to test for associations between generation and allele frequency in Generation Proxy Selection Mapping (GPSM). This generation proxy test was used to test for directional selection causing changes in allele frequency over time while accounting for population structure. We used submission year as the response variable in a GWAS model.

## Results

### Partitioning phenotypic variance

Using the NCPT data, we partitioned the genetic variance for yield into its constituent parts (Table S1). The different components of GxE (genotype-by-year, genotype-by-location, and genotype-by-year-by-location) explained a large proportion of the genetic variance (5.56%, 22.7%, and 34.1%, respectively). Genotype’s main effect explained just 27.6% of the genetic variance, which demonstrates the relative importance of GxE to yield in potato.

### Association with discrete program during selection

In total, twelve significant markers were identified across four selection sites (Maine, Michigan, New York, and Wisconsin) in the discrete program test (Table 3). All significant SNPs identified were unique within selection site (i.e., no overlapping markers were identified), though Maine and New York both had peaks on chromosome 4 within 2 Mb of one another. Linkage disequilibrium estimates in the US cultivated potato between 1 and 5 Mb are generally high (Pearson’s correlation =  ~ 0.1) depending on the population and chromosome of interest, and long-range linkage disequilibrium tends to decay more slowly than in diploid plants (Vos et al. 2017; Sharma et al. 2018). PCA on the genotypes showed little evidence for population structure (Figure S1), indicating that these associations were not likely due to population stratification between breeding programs. We calculated percent variance explained for significantly associated markers by backward elimination. The cumulative SNPs associated with Maine, Michigan, New York and Wisconsin explained 3.1%, 9.6%, 10.0%, and 2.8% of the variance, respectively. It is important to note that the percent variance explained may be inflated from the artificially reduced sample size when using a binary trait due to the Beavis effect (Beavis 1998).

We identified two SNPs associated with latitude and a single SNP associated with longitude (Table 4). Estimated effect sizes for latitude were 0.78 and 1.88 degrees latitude and explained 5.0% of the variance. The SNP associated with longitude had an estimated effect size of − 2.41 degrees longitude, explaining 2.6% of the variance. Estimated effect size refers to a change in the estimated latitude or longitude e.g., an estimated effect size of 2 degrees in the latitude model would mean a copy of the alternative SNP is associated with a selection site that is 222 km farther north than genotypes without a copy of the alternative SNP.

**Table 4:** GWAS results from latitude and longitude models. Chr: chromosome. Effect: effect size estimate. PVE: percent variance explained (%). Effect sizes are in degrees latitude/longitude

Trait	Marker	Chr	Position	LOD Score	Effect	PVE
Latitude	PotVar0044336	7	53,313,090	5.29	1.87	2.44
Latitude	solcap_snp_c1_8390	9	59,763,922	5.33	0.78	2.55
Longitude	ST4.03ch08_1934839	8	2,563,277	5.40	− 2.41	2.57

### Association with continuous environmental variables during selection

**Fig. 2:**
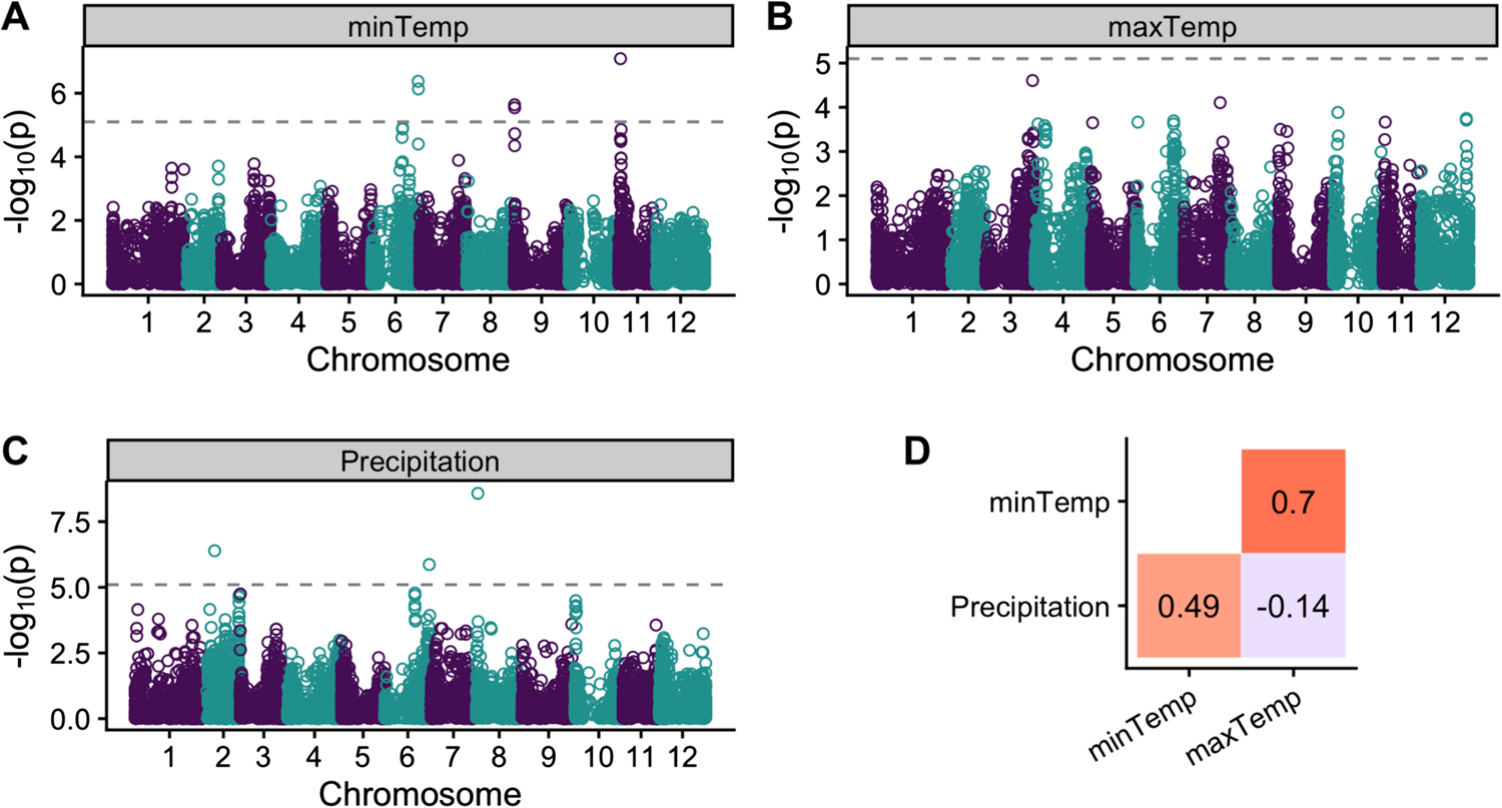
(**A**–**C**) Manhattan plots of envGWAS results for the different environmental variables during selection: **A** minimum daily temperature, **B** maximum daily temperature and **C** precipitation. Dashed lines represent the 5% significance threshold adjusted for the number of effective markers. **D** Pearson's correlations (*r*) between the different environmental variables at the selection locations

We identified six SNPs associated with two of the three environmental variables tested (minTemp and precipitation) (Table 5). For the minTemp model, we found three significant markers with effects ranging from –0.91 to 0.47 degrees C which explain 8.9% of the variance. In the precipitation model, we find three significant markers with estimated effect sizes of − 3.66 and 2.55 cm explaining 10.8% of the variance (Fig. 2C). There was a relatively strong positive Pearson’s correlation (*r*) between minTemp and maxTemp (0.70), a relatively weak negative *r* between maxTemp and precipitation (− 0.14), and a relatively strong positive *r* between minTemp and precipitation (0.49, Fig. 2D).

**Table 5:** GWAS results from selection environment (continuous environmental variable) models. Chr: chromosome. Effect: effect size estimate. PVE (%): percent variance explained. Effect sizes are degrees Celsius (maxTemp, minTemp) and cm (precipitation)

Trait	Marker	Chr	Position	LOD Score	Effect	PVE
minTemp	ST4.03ch06_57692614	6	57,440,194	6.38	− 0.92	2.71
minTemp	ST4.03ch09_411487	9	435,297	5.64	− 0.41	2.60
minTemp	solcap_snp_c1_11105	11	2,567,216	7.09	0.47	3.60
Precipitation	ST4.03ch02_14148961	2	13,661,372	6.39	2.55	2.82
Precipitation	ST4.03ch06_57244824	6	57,004,580	5.87	− 3.66	3.56
Precipitation	ST4.03ch08_1934839	8	2,563,277	8.58	− 2.85	4.43

### Association with regression of yield and environmental variables during the trial

**Fig. 3:**
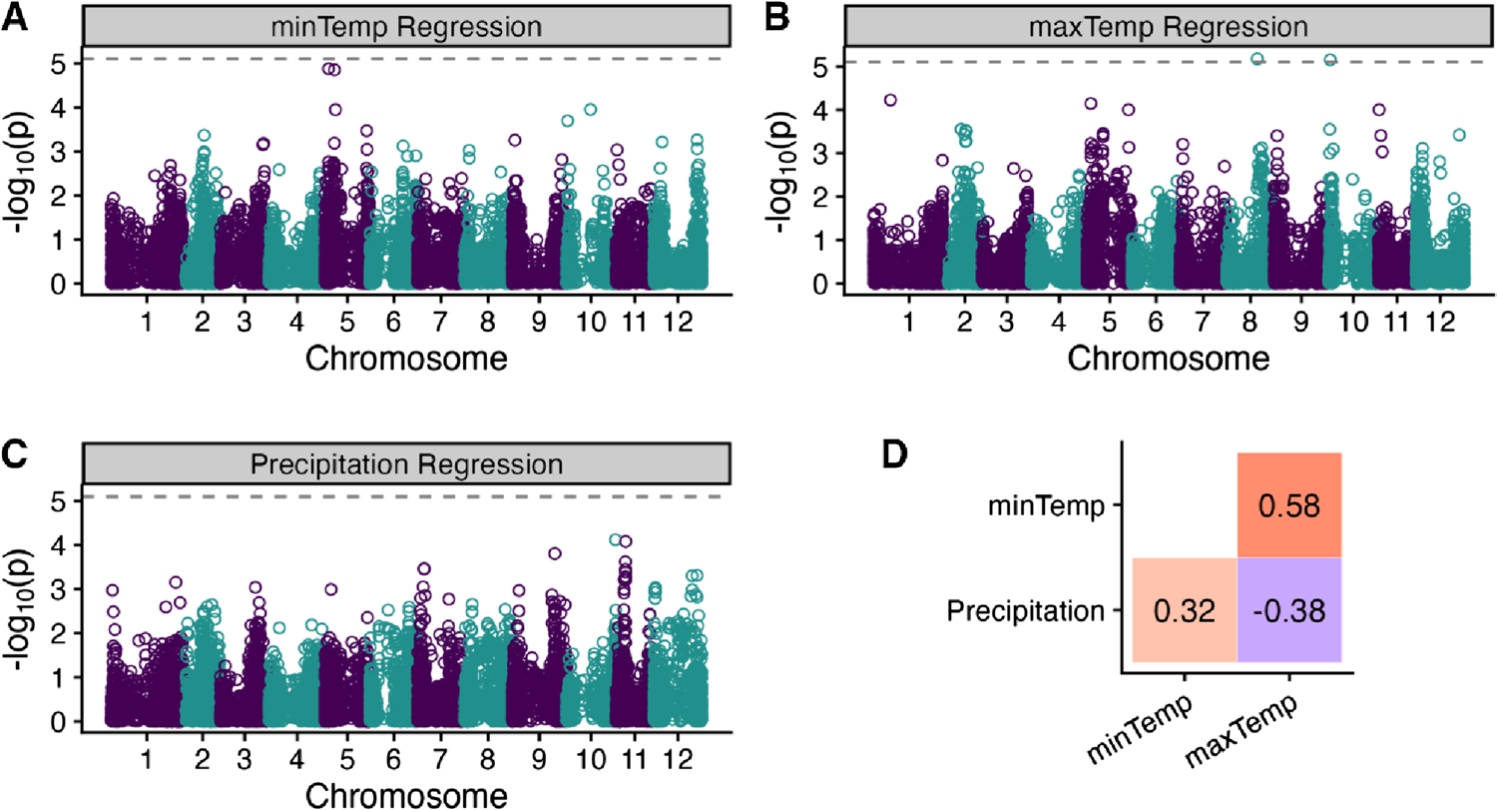
(**A**–**C**) Manhattan plots of regression GWAS for (**A**) minimum daily temperature, **B** maximum daily temperature and **C** precipitation at the trial locations. Dashed lines represent the 5% significance threshold adjusted for the number of effective markers. **D** Pearson's correlations (*r*) between the different environmental variables at the trial locations

To find SNPs that may be underlying GxE for yield across specific environmental gradients during the trial, we looked for associations between SNP frequencies and the slope of the regression line of yield onto three environmental variables in our regression GWAS models. We found two unique SNPs significantly associated in our regression GWAS for maxTemp, while no SNPs were identified in the precipitation or minTemp regression GWAS (Fig. 3 and Table 6). The markers identified in the maxTemp regression GWAS model explain 1.2% and 2.5% of the variance. There was a relatively strong positive *r* between minTemp and maxTemp (0.58), a moderate negative *r* between maxTemp and precipitation (− 0.38), and a moderate positive *r* between minTemp and precipitation (0.32, Fig. 3D).

**Table 6:** GWAS results from regression models. Chr: chromosome. Effect: effect size estimate. PVE (%): percent variance explained. Effect sizes refer to changes in estimated slope from the regression models used

Trait	Marker	Chr	Position	LOD Score	Effect	PVE
maxTemp slope	solcap_snp_c2_32802	8	38,420,172	5.18	0.012	1.40
maxTemp slope	ST4.03ch10_1779334	10	1,693,606	5.15	− 0.022	2.49

### Generation proxy selection mapping

**Fig. 4:**
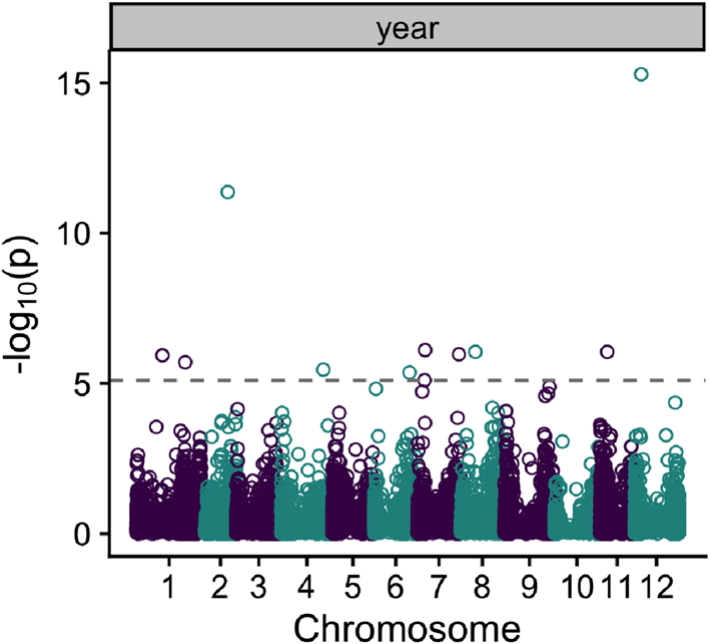
Manhattan plot of Generation Proxy Selection Mapping (GPSM) model. We use the year a clone was first entered into the national trial as a proxy for its generation. The dashed line represents the 5% significance threshold adjusted for the number of effective markers. Points above the dashed line represent SNPs that changed in frequency throughout the years tested

We tested for changes in allele frequency over time by Generation Proxy Selection Mapping (GPSM) to see if SNPs identified in our previous tests changed in frequency over the period investigated. We identified ten SNPs significantly associated with the year a clone was originally submitted to the NCPT (Fig. 4 and Table S2). No significant markers identified in the GPSM model were within 2 Mb of those identified in either the envGWAS models or regression GWAS, and only three markers were identified within 5 Mb (Table S2).

## Discussion

### Local adaptation is transient in the NCPT

We did not find evidence of directional selection for local adaptation in our GPSM model. There were no markers identified in our GPSM model within 1 Mb of either the markers identified by envGWAS or those identified by regression GWAS models. This result confirmed the expectation that local adaptation is transient in the NCPT. In other words, while GxE plays an important role in driving selection in early field years leading to local adaptation, only conditionally neutral alleles perform well across the trials and clones with broad application are overrepresented as parents. The selection of broadly adapted clones and the exchange of material between breeding programs means gene swamping is likely to prevent locally adapted alleles from rising in frequency. This is especially true in potato, where there is a very limited population structure and released lines are required to perform well widely. In other contexts, gene swamping may not suppress directional selection for local adaptation within a breeding program if a stronger within-program population structure exists and lines are expected to be grown over smaller geographic ranges.

### Breeding for ecotypes within potato

Presently, potato breeders in the US focus on releasing lines that perform well across broad geographic ranges in multiple production systems. One reason for this is chip processing plants accept only specific varieties in an attempt to reduce the phenotypic variation and waste from automation, with only coarse scale stratification (northern versus southern). The relative sparsity of associations with latitude and longitude we uncovered is consistent with the findings of previous studies (Schmitz Carley et al. 2019), which suggest that latitude is not sufficient to explain variance in potato-growing environments. Moreover, as the climate continues to change, growing a single variety across a large geographic range may lead to *more* phenotypic variation because of the large GxE component contributing to genetic variance in agronomically important traits.

Phenotypic variation could potentially be reduced by using ecotypes, lines selected for specific environments. Schmitz Carley et al. (2019) lay out the groundwork to breed for regionally adapted varieties by quantifying the genetic covariance of environments in the NCPT, i.e., quantifying the genetic response to environment between regions. This method highlights the similar effect of regions on genotypes without requiring the identification of the environmental factor responsible for that effect (climate, geography, soil type, production system, etc.). Knowing the genetic covariance of environments allows breeders to appropriately weigh the importance of phenotypes in each trial location to breed for a specific region. This work expands on that by identifying specific genomic regions and potential variants that may be important during ecotype establishment.

However, we suggest envGWAS and regression GWAS be used cautiously, as these methods cannot replace fine-scale mapping techniques to identify causal loci underpinning environmental adaptation. Further, it is important to note that the regression GWAS ignores the error in the slope estimate, which was considerable: 95% confidence intervals were large relative to the range of slope estimates, and many confidence intervals crossed zero (e.g., the confidence interval contained both positive and negative estimates for a genotype’s slope). We also assume a linear relationship between yield and environmental measurements, which is often not the case. We justify the use of regression GWAS by treating these errors as measurement error and expect such error to be randomly distributed between genotypes, which, while reducing power considerably, should not result in an excess of spurious correlations.

### Local adaptation influences early field year selection

We found several SNPs associated with both specific breeding programs and environmental variables during selection. Identified SNPs associated with the selection environment explain as much as 10.8% of the variance in environmental measurements, implying that selection is leading to an overabundance of these SNPs. This suggests that while local adaptation may provide a benefit within certain environments, it may hinder performance outside of that context. So, while there may be SNPs that are repeatedly selected within certain environments, they fail to rise in frequency in the greater population as a product of gene swamping during the national trial, as discussed above. The antagonistic process of selection within environment and subsequent gene swamping acts to slow breeding progress as the genetic gains within selection environment are not realized in the broader context. This further emphasizes the potential benefit of regional varieties. With regional variety development, producers and processors can take advantage of the genetic gains for beneficial environmental responses realized during selection.

Environment is not the only difference in selective pressure across breeding programs. Breeders focusing on one trait over another may also lead to associations that would not necessarily correlate with the environment, and, while breeder preference would still act to increase fitness of those genotypes with the more optimal trait value, this does not fit the traditional definition of local adaptation. Furthermore, while we found very little population structure among the NCPT genotypes, it is still possible that what structure is there is leading to spurious associations. Breeder preference and population structure are confounded with environment within the program specific GWAS models. However, there is no reason to believe that either of these would be correlated with environmental variables. We found no overlap between the significant markers identified in the program specific models and those identified by other envGWAS models. This suggests that the significant SNPs we find in the models for environmental variables are due to environmental response and not due to the idiosyncrasies that cause spurious associations in the case-control program specific models.

## Conclusions

While breeding efforts in potato often focus on identifying cultivars that perform well across a wide range of environments, early selection takes place in a relatively small region. Given the high proportion of phenotypic variance explained by GxE, this early breeding strategy should result in lines that perform best where they were selected i.e., locally adapted lines. To understand how this selection scheme impacts the frequency of SNPs associated with continuous environmental variables during early field year selection, we employed envGWAS to search for markers associated with both specific environmental variables and general environments during the first 3 years of selection. These tests resulted in the identification of many SNPs across the genome which may represent parts of the genome under selection for local adaptation. We also found SNPs associated with the regression between environmental variables at each growing location and yield. These SNPs may be indicative of QTL underlying a yield advantage across different environmental gradients. We did not find evidence of directional selection for SNPs underlying local adaptation, implying that local adaptation is transient under the current potato breeding strategy. This method can be applied to other species that collect phenotypic and genotypic data on individuals that were selected in different conditions and grown in common environments. These results can be used to generate hypotheses about the molecular basis of local adaptation/response to environment and to breed crops for specific environments by identifying markers and their effects across those environments. As the climate continues to change, moving breeding targets from varieties with broad application to ecotypes will become more important. Our results suggest that existing data can be used to jumpstart the process of identifying causal loci underlying environmental response for rapid ecotype development through marker-assisted selection and/or genomic selection.

### Supplementary Information

Below is the link to the electronic supplementary material.

Supplementary file1 (DOCX 1546 kb)

## Data Availability

Formatted data sets and scripts used for analysis are available at https://github.com/shannonlabumn/NationalTrialEnv
