# Supplementary material for: Genotype-by-environment interactions and local adaptation shape selection in the US National Chip Processing Trial
Source: Theor Appl Genet. 2024 Apr 10;137(5):99. doi: 10.1007/s00122-024-04610-3 (PMC11006776; doi:10.1007/s00122-024-04610-3)
Supplement: Supplementary file 1 — Supplementary file1 (DOCX 1546 kb) [file 122_2024_4610_MOESM1_ESM.docx]

Figure S1: Plot of the first two principal components from principal component analysis of the marker genotypes. The different colored points show the ten public breeding programs where the genotypes included in the analysis originated. Ellipses contain 68% of genotypes from each program. Breeding program ellipses were largely overlapping, and the first two principal components explain only a combined 7.5% of the variance in the genotypes, indicating little population structure among breeding programs.


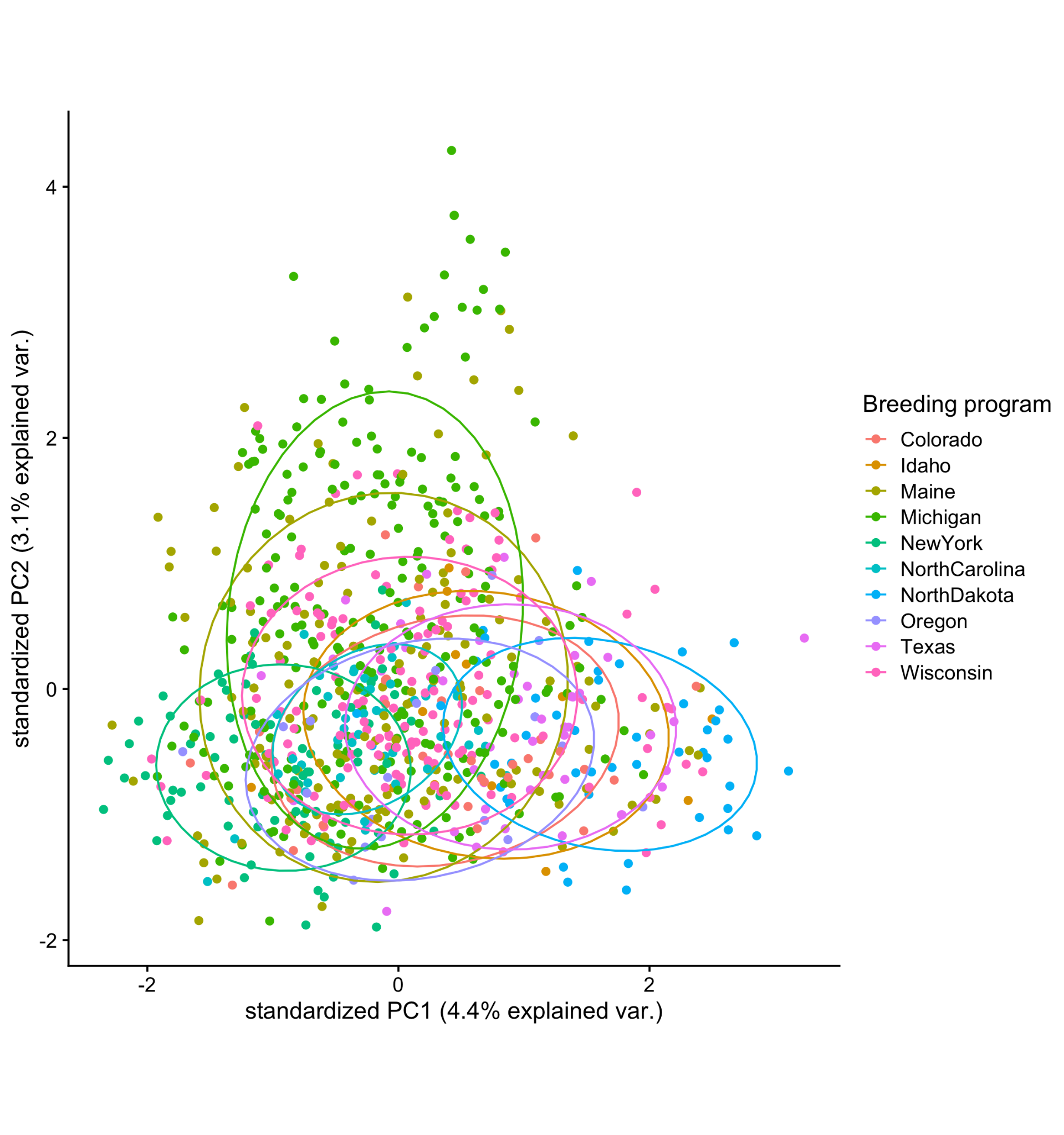


Figure S2: Manhattan plots of GWAS results for the discrete programs, (A) Colorado, (B) Idaho, (C) Maine, (D) Michigan, (E) North Carolina, (F) North Dakota, (G) New York, (H) Oregon, (I) Texas, and (J) Wisconsin. Dashed lines represent an adjusted significance threshold of p = 0.05.


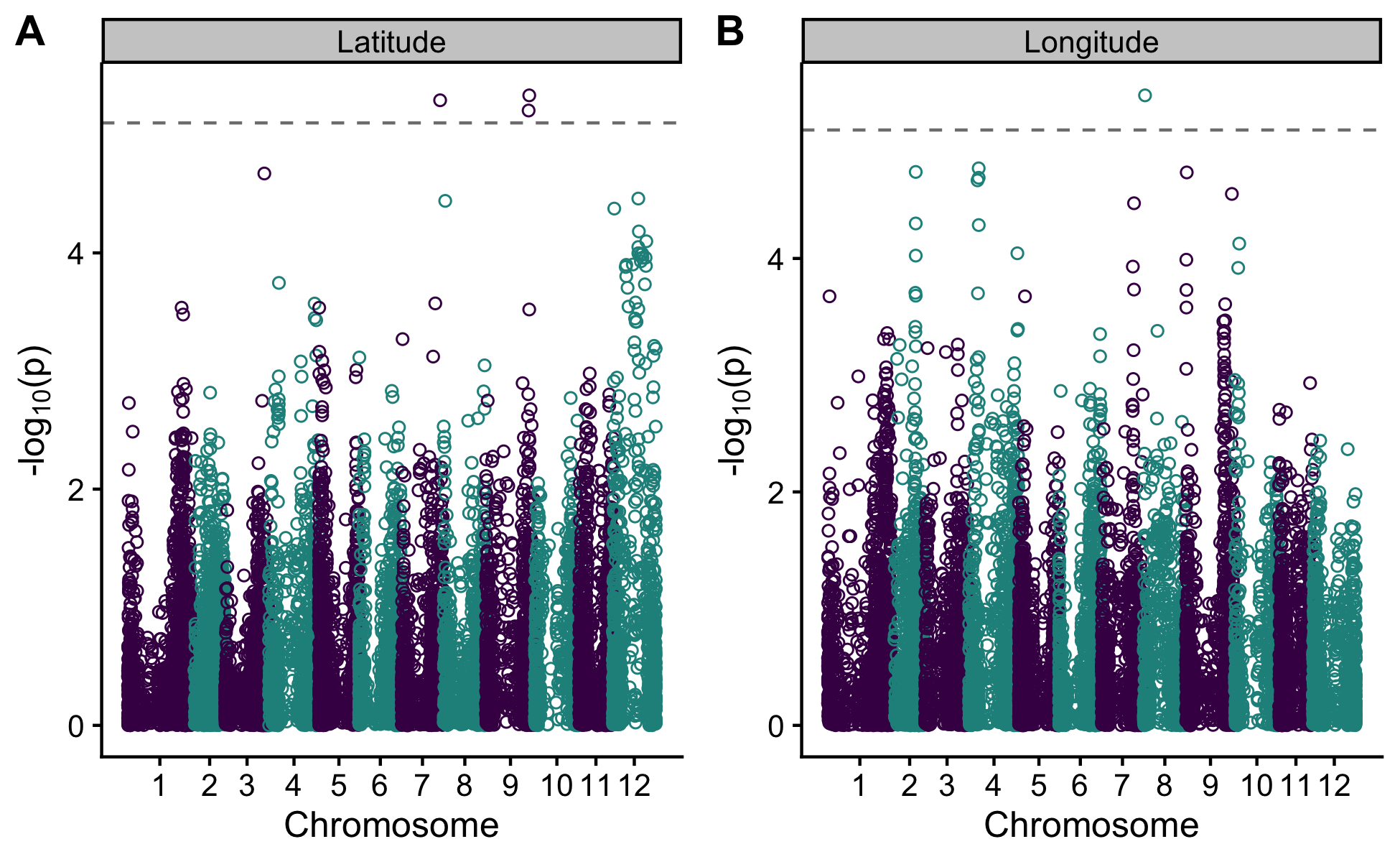


Figure S2: Manhattan plot of GWAS results for (A) latitude and (B) longitude. Dashed lines represent an adjusted significance threshold of p = 0.05.

Table S1: Genetic variance decomposition and percent variance explained (PVE) for yield data from all locations and years from 2010-2022.

| **Component** | **Variance** | **PVE** |
| --- | --- | --- |
| Genotype | 4.63 | 27.60 |
| Genotype-by-Year | 0.93 | 5.56 |
| Genotype-by-Location | 3.80 | 22.70 |
| Genotype-by-Year-by-Location | 5.71 | 34.10 |
| Residuals | 1.67 | 9.95 |

Table S2: Results from Generation Proxy selection mapping. Chr: chromosome. Effect: effect size estimate. PVE (%): percent variance explained. Effect sizes refer to generation (year) estimates.

| **Trait** | **Marker** | **Chr** | **Position** | **LOD Score** | **Effect** | **PVE** |
| --- | --- | --- | --- | --- | --- | --- |
| Year | solcap_snp_c2_34940 | 1 | 35398310 | 5.93 | -0.48 | 1.82 |
| Year | PotVar0033059 | 1 | 66102702 | 5.70 | 1.06 | 2.18 |
| Year | solcap_snp_c2_17807 | 2 | 34808312 | 11.37 | -0.79 | 2.40 |
| Year | solcap_snp_c1_10751 | 4 | 56641710 | 5.46 | -0.40 | 2.29 |
| Year | ST4.03ch06_48041419 | 6 | 48316731 | 5.36 | 0.81 | 2.07 |
| Year | solcap_snp_c2_6621 | 7 | 10250834 | 6.11 | -0.50 | 0.22 |
| Year | solcap_snp_c2_18548 | 7 | 55774840 | 5.97 | -0.65 | 1.96 |
| Year | solcap_snp_c1_6140 | 8 | 20286345 | 6.05 | -0.62 | 1.31 |
| Year | solcap_snp_c2_53685 | 11 | 10190018 | 6.05 | -0.63 | 1.38 |
| Year | solcap_snp_c2_18834 | 12 | 9100659 | 15.29 | -1.47 | 5.35 |
